# Using night time lights to find regional inequality in India and its relationship with economic development

**DOI:** 10.1371/journal.pone.0241907

**Published:** 2020-11-16

**Authors:** Abhishek Singhal, Sohini Sahu, Siddhartha Chattopadhyay, Abhijit Mukherjee, Soumendra N. Bhanja

**Affiliations:** 1 Department of Economic Sciences, Indian Institute of Technology Kanpur, Kanpur, Uttar Pradesh, India; 2 Department of Humanities and Social Sciences, Indian Institute of Technology Kharagpur, Kharagpur, West Bengal, India; 3 Department of Geology and Geophysics, Indian Institute of Technology Kharagpur, Kharagpur, West Bengal, India; 4 Interdisciplinary Centre for Water Research, Indian Institute of Science, Bengaluru, Karnataka, India; University of Western Australia, AUSTRALIA

## Abstract

Due to unavailability of consistent income data at the sub-state or district level in developing countries, it is difficult to generate consistent and reliable economic inequality estimates at the disaggregated level. To address this issue, this paper employs the association between night time lights and economic activities for India at the sub-state or district-level, and calculates regional income inequality using Gini coefficients. Additionally, we estimate the relationship between night time lights and socio-economic development for regions in India. We employ a newly available data on regional socio-economic development (Social Progress Index), as well as an index that represents institutional quality or governance. Robust to the choice of socio-economic development indicators, our findings indicate that regional inequality measured by night time lights follow the Kuznets curve pattern. This implies that starting from low levels of socio-economic development or quality of institutions, inequality rises as regional socio-economic factors or quality of institutions improve, and with subsequent progress in socio-economic factors or quality of institutions, regional inequality declines.

## Introduction

**Fig 1:**
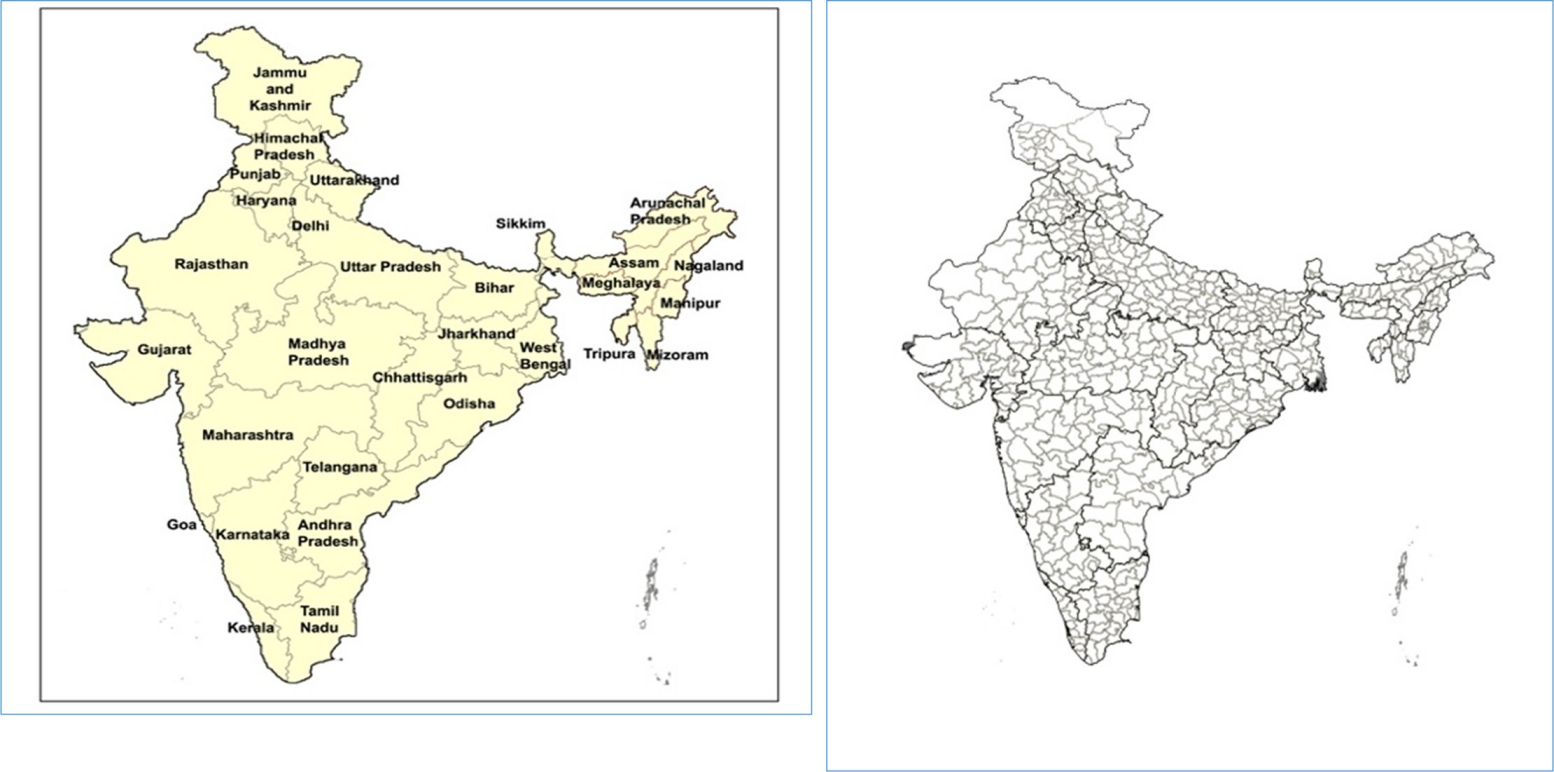
a. States of India. b. Districts of India. Source: Authors.

India is one of the fastest growing economies in the world. While per-capita income in India is gradually rising, the issue of distribution of income that is represented by income inequality has been somewhat side-tracked. Economic inequality has serious implications from the perspective of human well-being as it leads to poverty, lack of access to education and health facilities, increase in crime and corruption etc. According to a report (Top 10% earners have 55% of India's wealth. https://economictimes.indiatimes.com/news/politics-and-nation/top-10-earners-have-55-of-indias-wealth-report/articleshow/62178437.cms) income inequality in India has been increasing since the deregulation started in the 1980s, with only top 10 percent of labour force accounting for 55 percent of the national wealth in 2016. India has been trying to tackle this rising inequality with drives against corruption and tax evasions, and schemes meant to improve access to energy and finance for the poor. But for forming and implementing these ideas and schemes, the zones that require attention need to be identified. Due to India being exceedingly diverse, both geographically and demographically, this task becomes highly cumbersome. At the sub-national level, at present, there are 28 states in India while at the sub-state level there are 739 districts (see Fig 1A and 1B).

The government needs to identify the areas being affected the most by wealth imbalance, collaborate with state governments and then work for their betterment. But it is easier said than done. The estimation of income at the district-level is beset with the problems of availability of data as well as collection and analysis of information. The central and state governments need district-level income data for the purpose of planning and policy making. They need to know the level of economic activity in a region and living standards of people to measure the economic growth of a district and draw comparisons. These comparisons would then help the authorities to measure income disparities between districts and identify the backward areas which need the most attention. The available state-level data gives an average value of per capita income in the state, thus bypassing districts and regions where per capita values might be far below than the state average. These poverty-afflicted areas can only be identified when income data is available at disaggregated levels such as for districts, towns/cities, and villages. Additionally, these per capita income figures could be misleading since they emphasize on average income but neglect income distribution within the region. The presence of excessive regional inequality is a hindrance to sustainable growth and affects the stability in the society. It is associated with conflicts and civil unrest, thus weakening the public’s confidence in the administration. Whereas, if this regional inequality is identified, then according to Fields and Schulz (1980) [1] it could help the authorities in *“formulating developmental policies aimed at alleviation of poverty*, *gauging the degree of country’s labour*, *market integration*, *understanding the pattern of population movements*, *predicting future urbanization and characterizing the poor”*. Availability of this data at sub-national-levels might also encourage local authorities such as district collectorate offices, block development offices, panchayat samitis, and municipal corporations to formulate policies, conduct social drives and awareness campaigns and help in economic development of their regions.

Income inequality in India has been calculated on the basis of consumption data available at household and individual levels. But even in this era of advancements, these estimates are limited to country or state-levels, and are not available at district or regional levels in a stable and consistent manner. Due to the huge expenses involved, the data is collected every five or ten years and not on a regular basis. Other road blocks include lack of standardized national income accounting methods and reliable methods of data collection, inefficiency of surveyors, and the subjective responses of the respondents. Households which are better-off might under-report their expenditures and belongings whereas poor households might misreport them. Since the data is collected through surveys and the resources employed are limited, so the collection of data is both a time and resource consuming exercise. In India, this socio-economic data is collected quinquennially at the district-level by the National Sample Survey Organization (NSSO) and annually at pan-India level by National Accounts Statistics (NAS). Inadequate statistical infrastructure, lack of an equalized price index and lack of economic integration across regions pose considerable macro-level data collection-related challenges in developing nations, including India.

The data available poses another problem since it is mainly available at the country and state-levels and is unavailable at geographically disaggregated levels; and even if available, there is a large time lag involved before they are recorded and made available to economic accounting agencies. Along with under-reporting and misreporting of data, inappropriate sampling (the sample is unable to represent population), and inappropriate weighting (inaccurate weights are assigned to samples for calculating population averages) also pose serious issues for data processing. In addition, a range of estimations and interpolations need to be undertaken which further increases the time gap. Consequently, governments such as in India have to resort to quick estimates, provisional estimates, etc. before a final estimate is made. In such a situation the government needs to look for alternative parameters that could measure the magnitude of economic activities and could act as a proxy for GDP. One such parameter is night time lights (hereafter is it denoted as NTL). NTL data is recorded at highly disaggregated levels and can be made available to economic accounting agencies within a relatively shorter span of time and a little need to generate improved estimates.

In developing countries, major spectrum of activities is under the domain of unorganised sector, so the data on most of the transactions remains unrecorded or corrupted. It is unable to reflect the true picture of the size of the economy due to the presence of a large informal sector. Consequently, the governments in developing countries such as India has to resort to provisional and quick estimates before the final estimates could be made.

This paper tries to overcome this problem by using NTL as a proxy for the level of economic activity. One of the appealing features of night time lights is its availability at the most disaggregated geographical levels. The data is captured through night-time imagery satellite and focuses on establishing a relationship between the inequality indices calculated by income estimates and night time lights. It is measured on a 30 arc sec level, corresponding to roughly 1 square kilometre at the equator. This means that the NTL data could be used to measure economic activities at sub-national-levels which are not usually captured in national accounts such as provinces, districts, towns/cities and villages. The underlying hypothesis being that the light is as good a proxy for calculating regional inequality as it is for income. Thus using the NTL, a new inequality index could be created at granular administrative levels that could be used for designing and implementing policies.

The Gini coefficient is the most commonly used standard measure of economic inequality. So we try to build state-level Gini coefficients using district-level NTL data. But we also need to control for population, since higher population could also lead to higher values of night lights. So we use district-level population data as weights while calculating Gini coefficients by using the NTL data.

Our findings indicate that high levels of inequality at the regional level is mainly from those high-income regions. This leads us to investigate if there is a Kuznets curve-type relation by analysing the relation between inequality and economic development. In doing so, we analyse the relationship between the Gini derived from the NTL, and social progress; and the Gini derived from the NTL and the quality of governance(that is a proxy for the level of economic development) by considering quadratic functional forms. The intrinsic value of good governance and institutions is instrumental for economic growth and economic development and is a vehicle towards a uniform income distribution.

Bhandari and Roychowdhury (2011) [2] pointed out that since Indian subcontinent is highly diversified both geographically and demographically, a model only containing NTL and district-level GDP would not be sufficient and thus other control variables need to be incorporated. They used dummies, according to geographical location and level of economic activities and significance of districts and found that district-level GDP is significantly explained by the night lights in that area. Henderson, Storeygard and Weil (2012) [3] hypothesized that regions that tend to be highly lit are wealthier relative to regions that are less so. However, since income per capita is badly measured in developing countries, regional variation in night time lights per capita proved to be an ideal candidate for estimating and understanding regional inequality.

Development in human well-being is increasingly being recognized as indispensable in the movement towards a sustainable future. Ghosh, Anderson, Elvidge and Sutton (2013) [4] used NTL data to developed various globally consistent proxy measures of human well-being at sub-national-levels such as Night Light Development Index (NLDI). In an earlier research work by Elvidge, Baugh, Anderson, Sutton and Ghosh (2012) [5], they did a similar exercise and developed the NLDI as a simple, spatially explicit and globally available empirical measurement of human development build solely on night time lights data and population density. Bundervoet, Maiyo and Sanghi (2015) [6] used the NTL data for estimating economic growth at sub-national-levels in Kenya and Rwanda. The reasons were for communities to share national prosperity, policy interests, and helping private investors undertake investments. Due to lack of reliable and consistent sub-national income data, Mveyange (2015) [7] estimated the regional income inequality in Africa using the NTL data and found a significant positive association between income and regional inequality. In a recent work by Chakravarty and Dehejia (2017) [8], they used NTL at the district level in India to estimate the convergence in income across states. They found that post-1991, states across India have exhibited divergence, thereby indicating rising income inequality across regions.

As far as the relation between inequality and economic development or quality of governance is concerned, Ezcurra and Pose (2014) [9] examined the relationship between government quality and spatial inequality across forty-six countries and found a negative and significant association between government quality and magnitude of regional disparities. Zhuang, Dios and Martin (2010) [10] investigated the role of governance and institutions in supporting growth in developing Asia. They argued that there is a positive association between good governance and the level of development. For both the above studies the authors used the same factors to measure the quality of governance namely voice and accountability, political stability & absence of violence, government effectiveness, regulatory quality, rule of law, and control of corruption. In lines of these variables used to measure quality of governance, we employ the Social Progressive Index (SPI) that captures the progress in India based on various social dimensions and later construct a Quality of Governance Index (QGI).

## Methods

### Input datasets

The US Air Force Defense Meteorological Satellite Program (DMSP) has designed the Operational Linescan System (OLS) sensors to continuously monitor the global-scale cloud cover, cloud top temperatures and night time satellite coverage. National Oceanic and Atmospheric Administration (NOAA) based National Geophysical Data Center (NGDC) started processing the nightlight luminosity data that originated from human activities. Luminosity signals from moonlight, clouds, seasonally late sunsets and auroral events were filtered out to prepare the dataset (https://ngdc.noaa.gov/eog/gcv4_readme.txt last accessed on September 25, 2020). The available non-cloudy, filtered observations available during an individual year are averaged and transformed to integers with values between 0–63 and termed as "digital numbers". Here '0' representing no lighting condition and '63' is the maximum possible lighting condition. The processed data was released globally at a spatial resolution of less than 1 km (30 arc-second) at an annual-scale between the years 1992 and 2013 (https://ngdc.noaa.gov/eog/dmsp/downloadV4composites.html last accessed on September 25, 2020). The data uniquely allow users to use nightlight luminosity information for estimating economic activities at a high spatial scale (sub-state level or more) [5]. As a result, several studies have used this data to investigate economic activities (Chen and Nordhaus (2011) [11], Henderson et al., (2012) [3], Michalopoulos and Papaioannou, (2013) [12], Pinkovskiy and Sala-i-Martin (2016) [13], Jean et al., (2016) [14]). The data has acquired for the Indian region at the native resolution between 1992–2013 and processed at the administrative block level through spatial averaging (number of blocks used = 7010).

**Fig 2:**
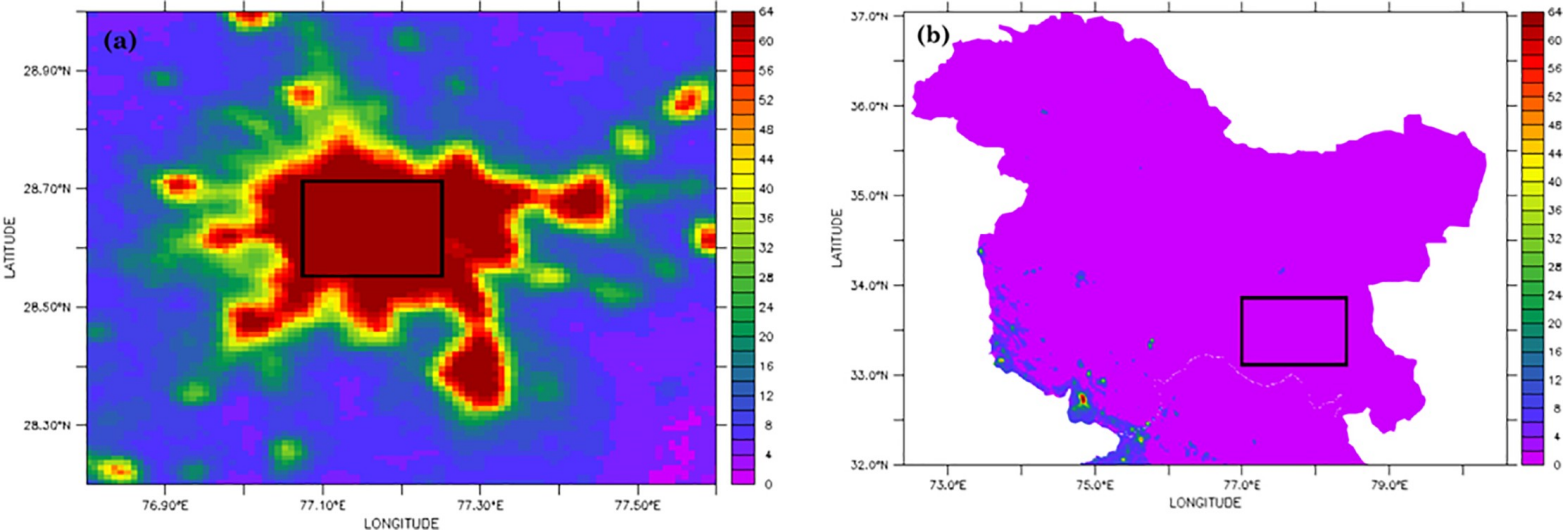
Maps of satellite-based nightlight digital numbers at (a) New Delhi in 1992 and at (b) parts of Himalaya in 1992. Digital numbers within the marked area are used for further analyses. Source: Authors.

Calibration between the inter-satellite measurements are not possible to conduct as the exact radiance values are not present (Hsu et al., 2015 [15]). We have performed the intercalibration in megacity New Delhi (Fig 2A) and parts of Himalaya (Fig 2B) following the approaches of Elvidge et al. (2009) [16] and Hsu et al. (2015) [15] between 1992 and 2013.

While the citycentre of New Delhi is believed to be developed from the start of the DMSP measurements in 1992, the Himalayan region is away from the development activities. As a result, we hypothesized that the digital numbers would be the highest (i.e. 63) in New Delhi and lowest (i.e. 0) in Himalaya. In order to check the performance of the multiple DMSP satellites, we have investigated the annual change in digital numbers from different satellite-based measurements between 1992 and 2013. Our analyses show almost no inter-annual change in the digital numbers obtained from the different satellites during the study period.

**Fig 3:**
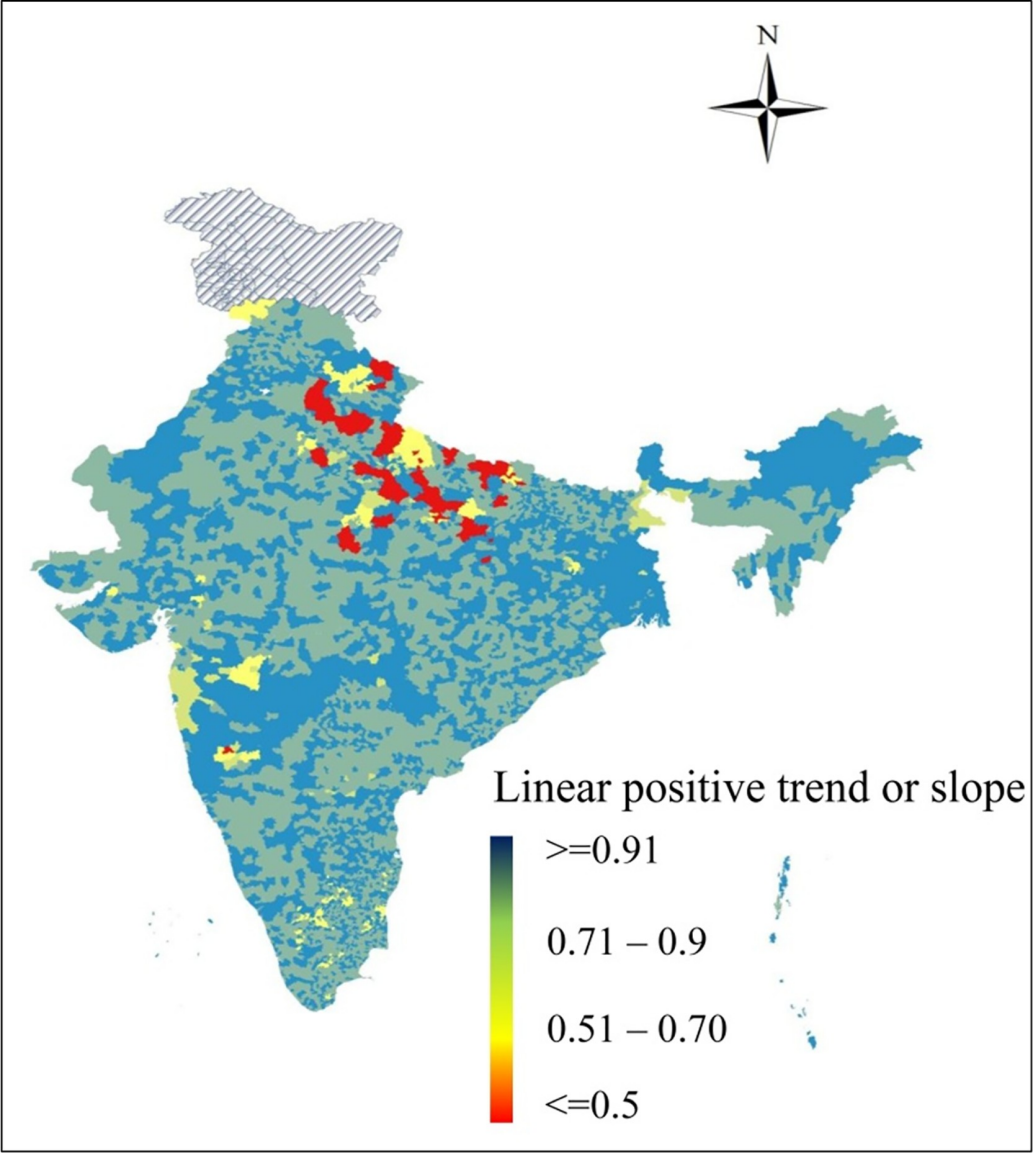
Annual linear trend of satellite based nightlight for 7010 administrative blocks for 1992–2013. Source: Authors.

The resulting set of filtered data for the study period is used for estimation purpose and the result is plotted in a map (Fig 3) using the ARC GIS (10.2).

The data for district-wise gross domestic product and population has been taken from the website of Planning Commission, Government of India, under state plans. The data is available sector-wise for both current prices and at the base price for the year 1999. We use the total District Domestic Product (DDP) data at constant prices of 1999–2000. The data is available for 23 Indian states, namely, Andhra Pradesh, Arunachal Pradesh, Assam, Bihar, Chhattisgarh, Haryana, Himachal Pradesh, Jharkhand, Karnataka, Kerala, Madhya Pradesh, Maharashtra, Manipur, Meghalaya, Mizoram, Orissa, Punjab, Rajasthan, Sikkim, Tamil Nadu, Uttar Pradesh, Uttarakhand and West Bengal, and for a period of ten years 1999–2008 (different for different states). The data for Gini coefficient that is based on consumption data, is the one that has been constructed by Das, Sinha and Mitra (2010) [17].

Data for social progress of India is provided by Social Progress India and is for a period of 8 years (2008–2012). The data is available for all Indian states and Delhi for all 12 factors (3 dimensions having 4 factors each). We use the data required for our analysis for generating the Social Progressive Index (SPI) and Quality of Governance Index (QGI).

We leave out Telangana from our analysis, as it was awarded the status of separate state in June 2014. Since it was a part of Andhra Pradesh, therefore all the data available prior to 2014 relates to the entire region covering both the states.

### Model selection

Following the methodology used by Bhandari and Roychowdhury (2011) [2], we first find the relationship between Night Time Light values (NTL) and Gross Domestic Product (GDP) for each district and see if NTL could be used as a proxy for GDP values. We estimate a linear regression on the pooled data of all districts in India. But since India is highly varied both geographically and demographically, using a model with only GDDP, NTL and population might not reveal the true picture. Thus, we use dummy variables for districts that might be outliers from the regular relationship between due to any reason. These dummy variables take the value 1 when the condition is satisfied by the district, else its value is 0. The categorization of districts based on the various dummy variables follow from Bhandari and Roychowdhury (2011) [2]. The regression model is given by Eq (1),

$$Ln\;GDDP=\beta_{0}+\beta_{NTL}Ln\;NTL+\beta_{Pop}Ln\;Pop+\beta_{Metro}D_{Metro}+\beta_{Submetro}D_{Submetro}+\beta_{Capital}D_{Capital}+\beta_{LargeCity}D_{LargeCity}+\beta_{Snow}D_{Snow}+\beta_{Forest}D_{forest}+\varepsilon$$ (1)

Where LnGDDP=NaturallogarithmofDistrictDomesticProduce

LnPop=Naturallogarithmofpopulationofthedistrict

LnNTL=NightTimeLightsvalueforthedistrict

$$D_{Metro}=Dummy\;for\;the\;district\;having\;a\;metropolitan\;city$$

$$D_{Submetro}=Dummy\;for\;the\;suburban\;areas\;to\;metropolitan\;cities$$

$$D_{Capital}=Dummy\;for\;the\;districts\;having\;state\;capitals$$

$$D_{LargeCity}=Dummy\;for\;the\;cities\;having\;high\;degree\;of\;economic\;activity$$

$$D_{Snow}=Districts\;having\;regions\;covered\;in\;snow$$

$$D_{forest}=Districts\;having\;large\;proportions\;of\;area\;covered\;by\;forests$$

For forest covered regions we did not have district-specific data. We took D_forest_ = 1 for all the states having average forest cover more than forty-five percent of total geographical area as per the Indian State of Forest Report, Forest Cover 2017. These states are Andhra Pradesh, Kerala, Manipur, Meghalaya, Mizoram, Sikkim and Uttarakhand.

Next, we go a step further and try to see if we can use NTL values instead of GDDP values for calculating the regional income inequalities. We follow the procedure given by Creedy (2015) [18] for computing Gini coefficients using weighted data. According to this work, for income level *x*_*i*_, for *i* = 1,2,3…,*n*, that has been arranged in ascending order (strictly non-decreasing) and each *x*_*i*_ has an integer weight *w*_*i*_, we can calculate the Gini coefficient by the following method.

$$Let\;N=\overset{n}{\underset{i=1}{\sum }}w_{i}$$ (2)

$$\&\;\bar{x}=\frac{1}{N}\overset{n}{\underset{i=1}{\sum }}x_{i}w_{i}$$ (3)

Where $\bar{x}$ is the weighted mean of all *x*_*i*_. In our case the *x*_*i*_’s are the Gross District Domestic Produce or the NTL value for each district *i* and the weights *w*_*i*_ are the population of the corresponding district *i*. Consequently, our *N* becomes the state population for the given year. We now define a new parameter *D*_*i*,*j*_ as follows. For *i* = 1 and *j* = 1,2,3,…,*w*_1_:

$$D_{i,j}=N+1-j$$ (4)

And for *i* = 2,3,…,*n* and *j* = 1,2,…,*w*_*i*_

Then:

$$D_{i,j}=N+1-\overset{i-1}{\underset{k=1}{\sum }}w_{k}-j$$ (5)

$$Gini=1+\frac{1}{N}-\frac{2}{N^{2}\bar{x}}\overset{n}{\underset{i=1}{\sum }}x_{i}\left(\overset{w_{i}}{\underset{j=1}{\sum }}D_{i,j}\right)$$ (6)

The population weighted Gini coefficient given in Eq (6) lies between *0* and *1*. It is intuitively appealing too as states with higher population is given relatively more weight than the states with lower population in the calculation of the Gini coefficient given in Eq (6). From the above methodology, we calculate two types of Gini coefficients: *Gini*_*NTL*_ & *Gini*_*GDDP*_, for individual years for respective states and estimate the corresponding regression models in Eqs (7) and (8) respectively.

$$Gini_{Consumption}=\alpha_{0}+\alpha_{NTL}Gini_{NTL}+\varepsilon_{0}$$ (7)

$$Gini_{GDDP}=\gamma_{0}+\gamma_{NTL}Gini_{NTL}+\varepsilon_{1}$$ (8)

Where,

$$Gi{ni}_{NTL}=Gini\;coefficient\;calculated\;on\;Night\;Time\;Lights\;values$$

$$Gini_{GDDP}=Gini\;coefficient\;calculated\;on\;Gross\;District\;Domestic\;Produce\;values$$

We further extend our research to find the relationship between the *Gini*_*NTL*_ and economic development to check if there is a Kuznets Curve-type relation which is common is studies involving inequality. We denote economic development through social progress and quality of governance at the state level. For this purpose, we use the Social Progress Index (SPI) for states made available by the Institute for Competitiveness, India (Kapoor, Kapoor and Krylova; 2017) [19]. Zhuang, Dios and Martin (2010) [10] and Ezcurra and Pose (2014) [9] in their studies for quality of institutions and its effects on income inequality has come up with six major dimensions that account for quality of governance namely,

1. Voice and accountability: measured by citizens’ perception of the extent of their role in selecting the government, having freedom of expression, association and free media.
2. Political stability and absence of violence: measured by the likelihood that the government would be destabilized or overthrown by unconstitutional and violent means including terrorism.
3. Government effectiveness: measurement of quality of public and civil services and their degree of independence from political pressure, quality of policy formulation, implementation and government’s commitment towards them.
4. Regulatory quality: measurement of ability of government in providing sound policies and regulatory framework that promotes private sector development.
5. Rule of law: measured by the limit to which the agents have confidence in and abide by the rules governing the society and contract enforcement. The spectrum includes property rights, police, courts and the risk of crimes.
6. Control of corruption: measured by the degree to which public power is exercised for private gain, including both petty and grand forms of corruption, as well as ‘capture’ of the state by elites and private interests.

We looked for related data for the above dimensions in the data available from Social Progress India and found the following relatable factors,

1. Personal safety: measures the extent of citizens’ safety from road deaths and violent crimes such as murder and rape.
2. Personal rights: measures if people are able to exercise their rights such as property rights, rights against human trafficking and judiciary.
3. Personal freedom and choice: measures if people are free of restrictions on their personal decisions, by looking for prevalence of corruption, child labour, early marriages and family planning.
4. Inclusion: measures the extent of opportunities each citizen gets for making his/her contribution to the society irrespective of age, sex or caste.
5. Access to advanced education: measures if people get opportunities for attaining higher levels of education through enrolment ratio and number of technical institutes and colleges.

Assuming all the above factors to be of equal importance we find the arithmetic mean of data values for above factors and develop a “Quality of Governance Index” (QGI). Finally, we try to find the relationship between *Gini*_*NTL*_ *and SPI*, and, *Gini*_*NTL*_ *and QGI*, respectively by employing OLS on the pooled data. We construct a quadratic model, since we check if there is a Kuznets curve-type relation, as given by Eqs (9) and (10).

$$Gini_{NTL}=\beta_{2}+\beta_{S\mathrm{PI}}SPI+\beta_{S\mathrm{PI}\;\mathrm{Square}}SPI^{2}+\varepsilon_{2}$$ (9)

$$Gini_{NTL}=\beta_{3}+\beta_{QGI}QGI+\beta_{QGI\mathrm{Square}}QGI^{2}+\varepsilon_{3}$$ (10)

## Results

### Simple correlations

**Fig 4:**
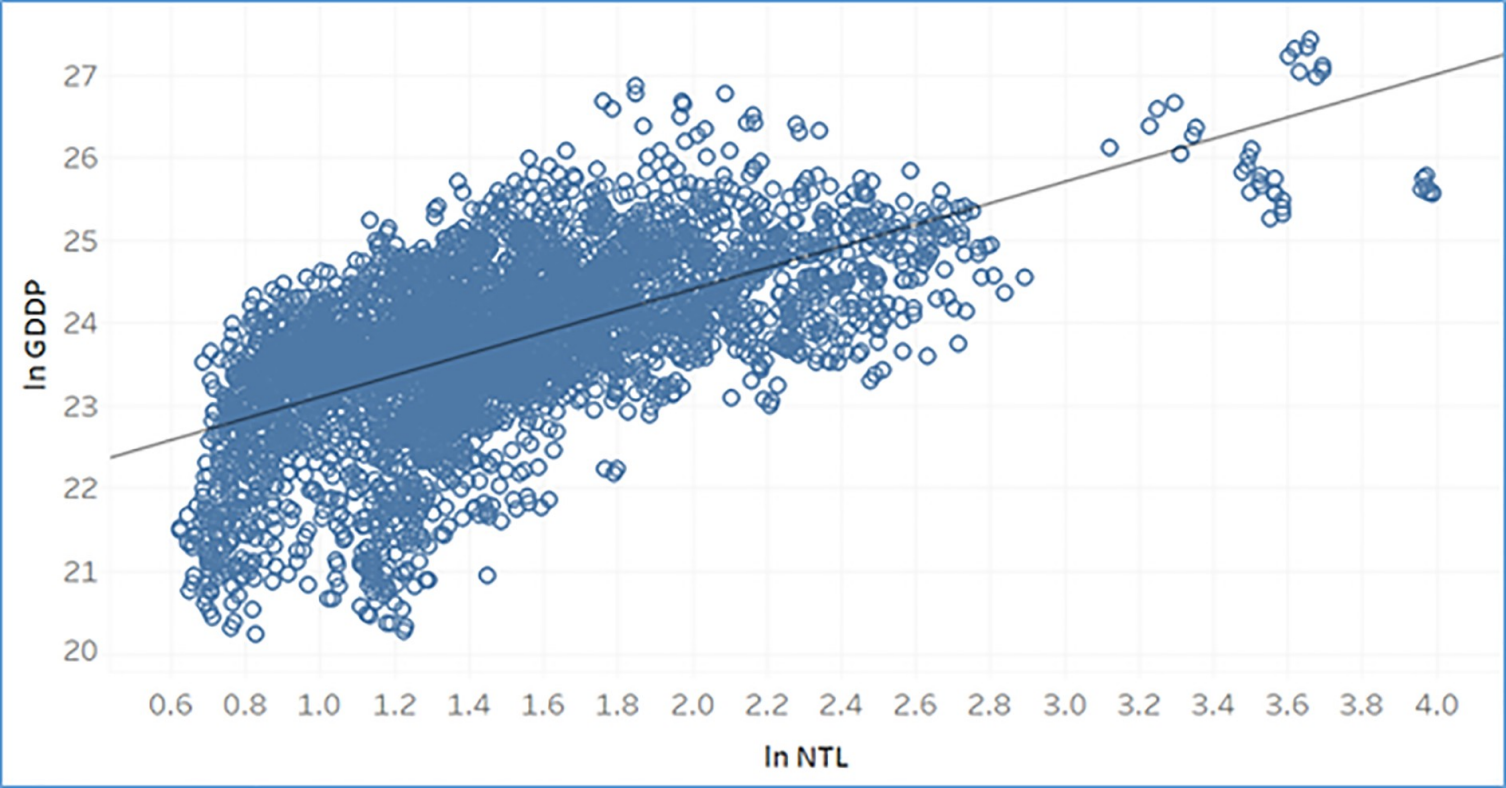
Scatter plot for gross district domestic product and night time lights for years 1999–2008. Source: Authors.

Following Mveyange (2015) [7], the link between economic activity in a region and how it leads to regional inequality can be explained as follows. Higher levels of economic activity in a region leads to higher income creation. Large proportions of this income couldn’t be recorded earlier due to them being under informal sector or businesses not disclosing their true incomes. But proportional parts of activities are undertaken during night hours as in during day hours, and they need to use lights. This light intensity is recorded, and true income estimates are generated. According to the intensity of light the regions could be brightly lit or poorly lit which further labels them as more wealthy areas or less wealthy areas. This generates income disparity between regions and leads to regional inequality. The trend for scatter plot between GDDP and NTL suggests there is a monotonic relationship between them (see Fig 4).

From the figure it is observed that as the value for GDDP increases, the corresponding value of NTL also increases and vice-versa.

### Regression analysis

*We performed the unit root tests and found all the variables to be stationary. On performing pooled OLS regression for gross district domestic produce (GDDP) and night time lights (NTL) (following Eq (1)), we obtained the results outlined i*n Table 1.

**Table 1:** OLS regression results for GDDP and NTL.

Coefficient	Value
ln(NTL)	0.53***
	(0.01)
ln(Population)	0.87***
	(0.01)
Metro	0.09
	(0.10)
Sub-metro	0.50***
	(0.04)
Capital	0.15***
	(0.03)
Large City	0.25***
	(0.02)
Snow	0.30***
	(0.03)
Forest	0.12***
	(0.03)
Constant	10.64
	(0.14)
*Adjusted R*^2^	0.87
No. of Observations	3819

Standard errors are in parenthesis.

Significance levels: *p < 0.1, **p < 0.05

***p < 0.01

From Table 1 we observe that GDDP and NTL are positively related, and all independent variables are statistically significant, except for the dummy for metropolitan districts. It could be accounted to the drawbacks of using night time lights. Metro cities are densely populated, but the city space is limited, so people live and work in close quarters in apartments and skyscrapers. Thus, the horizontal spread of lights is converted to proportional vertical spread, and so the illuminance captured by the satellite sensors is only a part of the actual illuminance. The high adjusted *R*^*2*^ indicate that about 87% of variability of GDDP can be explained by the NTL and other factors represented by the various dummy variables. Next, we regressed *Gini*_*Consumption*_ on *Gini*_*NTL*,_ following Eq (7) (see Table 2), and *Gini*_*GDDP*_ on *Gini*_*NTL*_, following Eq (8) (see Table 3).

**Table 2:** OLS regression results for *GINI*_*Consumption*_ with *GINI*_*NTL*_.

Coefficient	Value
*GINI*_*NTL*_	0.20***
	(0.04)
*Constant*	0.21
	(0.01)
*Adjusted R*^2^	0.13
No. of Observations	89

Standard errors are in parenthesis.

Significance levels: * p<0.1, ** p<0.05

*** p<0.01

**Table 3:** OLS regression results for *GINI*_*GDDP*_ with *GINI*_*NTL*_.

Coefficient	Value
*GINI*_*NTL*_	0.33***
	(0.07)
*Constant*	0.25
*Adjusted R*^2^	0.3471
No. of Observations	60

Standard errors are in parenthesis.

Significance levels: * p<0.1, ** p<0.05

*** p<0.01

It is observed that *Gini*_*NTL*_ is positively related with both *Gini*_*Consumption*_ and *Gini*_*GDDP*_, and the relationship is significant. This strengthens our hypothesis that inequality estimates calculated using NTL could be used as regional inequality estimates when the data for GDDP or consumption is unavailable. The low adjusted *R*^*2*^ leaves room for covering proportions of the dependent variable (*Gini*_*Consumption*_ *and Gini*_*GDDP*_) which *Gini*_*NTL*_ is unable to explain and thus other factors could also be brought into the picture (such as access to education, mean life expectancy, etc.) and the adjusted *R*^*2*^ could be improved upon.

Next, we explain the results when a Kuznets curve-type relation is tested between NTL-based inequality and development proxies, like the social progress index (SPI), and the quality of governance index (QGI), that has been explained in details in Model selection.

From Table 4 (follows from Eq (9)) and Table 5 (follows from Eq (10)) we observe that *Gini*_*NTL*_ follows a quadratic relationship with both SPI and QGI.

**Table 4:** OLS regression results for *GINI*_*NTL*_ with *SPI* and *SPI*^*2*^.

Coefficient	Value
*SPI*	0.0982***
	(0.0136)
*SPI*^*2*^	-0.001***
	(0.0001)
*Constant*	-2.1558
	(0.14)
*Adjusted R*^2^	0.3993
No. of Observations	60

Standard errors are in parenthesis.

Significance levels: * p<0.1, ** p<0.05

*** p<0.01

**Table 5:** OLS regression results for *GINI*_*NTL*_ with *QGI* and *QGI*^*2*^.

Coefficient	Value
*QGI*	0.0729***
	(0.1071)
*QGI*^*2*^	-0.0008***
	(0.0001)
*Constant*	-1.3230
	(0.14)
*Adjusted R*^2^	0.3471
No. of Observations	60

Standard errors are in parenthesis.

Significance levels: * p<0.1, ** p<0.05

*** p<0.01

**Fig 5:**
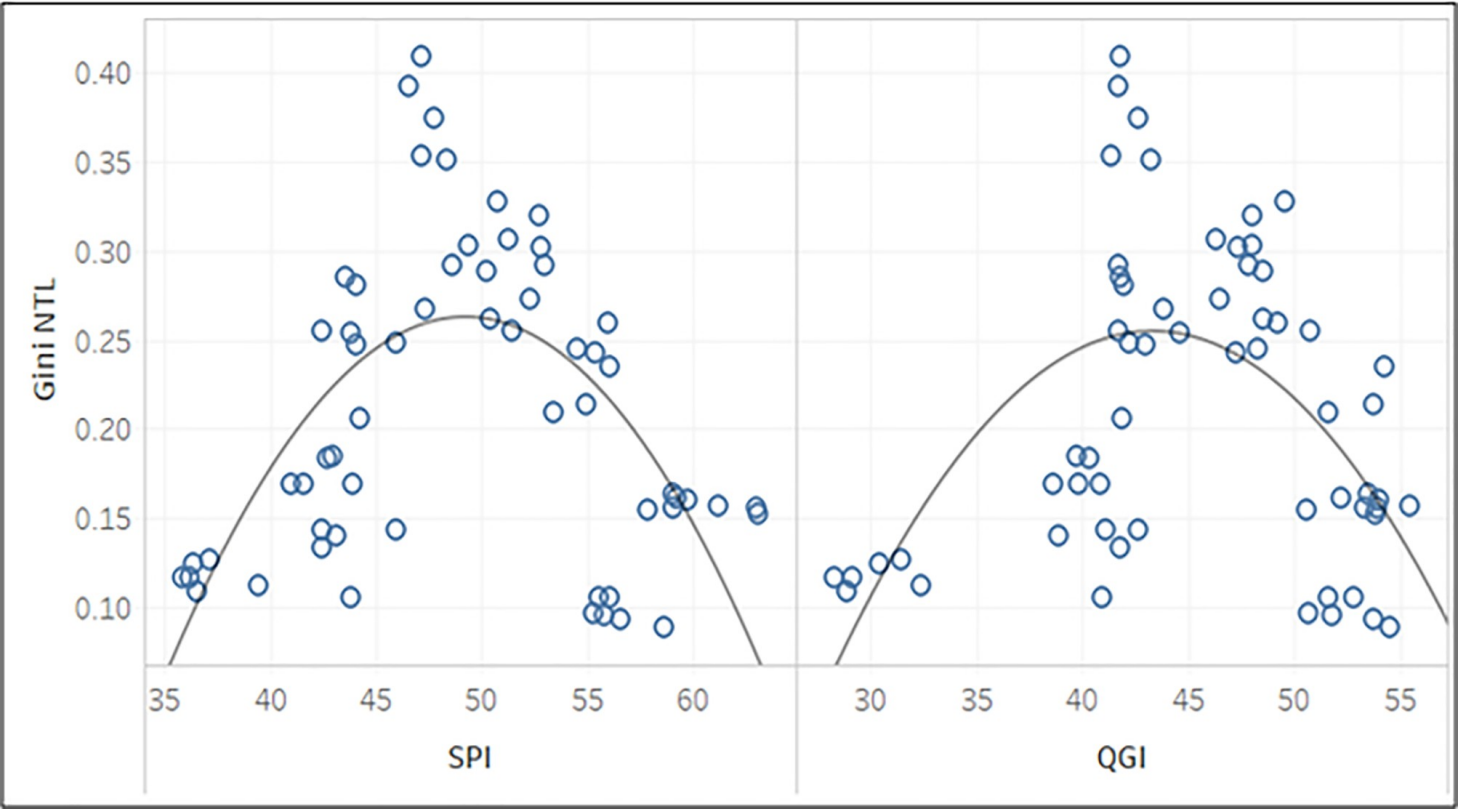
Trends between *Gini*_*NTL*_ and SPI; *Gini*_*NTL*_ and QGI.

This is also represented in Fig 5, where we observe that SPI and QGI both have an inverted-U shaped relation with respect to *Gini*_*NTL*_.

This indicates that as SPI or QGI increase, inequality increases initially, then reaches the maximum point, and thereafter it falls.

## Discussion

We categorize the *Gini*_*NTL*_ in 5 groups according to its severity, and obtained the heat maps. The grouping is done as follows:

$$Very\;Low\;=\;Gini_{NTL}<0.05$$

$$Low\;=\;0.05=<Gini_{NTL}<0.15$$

$$Moderate\;=\;0.15=<Gini_{NTL}<0.25$$

$$High\;=\;0.25=<Gini_{NTL}<0.35$$

$$Very\;High\;=\;0.35=<Gini_{NTL}$$

**Fig 6:**
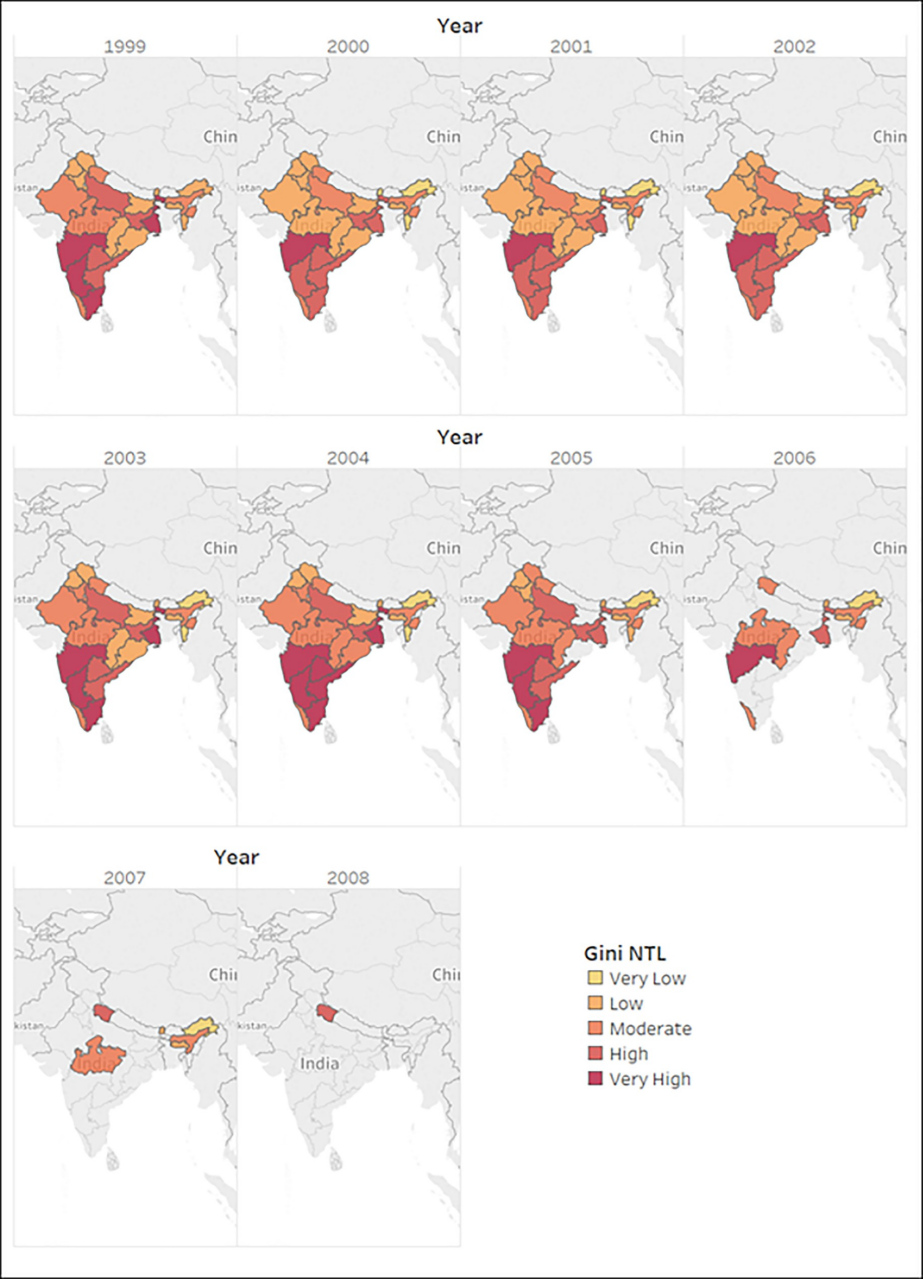
Gini_NTL_ heat map for Indian states for 1999–2008. Source: Authors.

Fig 6 shows the heat map for *Gini*_*NTL*_ for Indian states for the period of 10 years (1999–2008). However, the data was unbalanced, so the data for complete 10 years was not available for all the states.

From Fig 6 we observe that inequality has been more prevalent in southern and the western parts of India as compared to the rest of India. But we also know that these are the same parts that have shown the largest domestic produce growth rates. This could be regarded to the Kuznets hypothesis for economic development which says that as development occurs, inequality first increases till it reaches a certain limit and then decreases. Since India is yet to become a developed nation, the Kuznets curve may be the answer for the prevalence of higher income inequality among the most fast-paced growing states. Since SPI and QGI are both dimensions of economic development, it again supports the possibility of our previous hypothesis with the heat map of *Gini*_*NTL*_ that economic development in India might be following the Kuznets curve. According to Kuznets, at early stages of development, income inequality increases as the economy grows. This is explained by the structural changes in the economy with the share of industrial sector increasing and agricultural sector decreases (in terms of output and employment). An agrarian economy (rural spaces) is assumed to have low inequality as compared to an industrial economy (urban spaces), so when people move from rural to urban spaces, at an aggregate level the country experiences a rise in inequality and this process continues till an equilibrium level is attained. Moreover, differences in income levels in agriculture and industry and access to technical advancements also contribute towards this rising inequality. As an economy adopts new technical advancements, these are first adopted by large-scale industries thereby increasing their output and income whereas the small-scale industries keep struggling to compete with those technically advanced goods. This continues until the time the technology is available for all, and everyone gets equal opportunities, that is when inequality begins to fall.

### Caveats about nightlights as a proxy for economic activity and inequality

Although NTL has been used extensively in the literature as a substitute for economic activity, some caveats about the limitations of this data is worth mentioning.

To begin with, nightlights data is recorded by capturing the emissions from open spaces such as highways, parks and open parking lots etc., but the light from the spaces inside the buildings cannot be captured efficiently, leading to biased results on the current workforce inside the building or unclear estimation of goods and services produced inside a factory. NTL data is also not able to distinguish between the lights emitted from skilled workforce from corporate offices, unskilled labor from industries and domestic habitations.

From the studies conducted in the past [2] some other limitations regarding linearity of the model used to explain the correlation between GDP and night time lights are also observed. Metropolitan cities and their satellite cities or adjoining suburban areas have high levels of economic activity which the NTL data is unable to explain. Other cities which may again have higher levels of GDP but is not reflected by their light output can be the cities with great industrial significance, with high levels of manufacturing output. Other outliers to this NTL and GDP relationship include areas with high vegetation cover such as forests and regions with harsh climatic conditions such as snow-capped peaks and deserts. Cantonments, defense & military regions also do not add much value to GDP as compared to the amount of light output. Tourism is yet another such industry, which generates less than proportional value to GDP as compared to the significant amount of generated night lights. On the contrary, shadow economy might contribute significantly to the value added in GDP if they are accounted for, but most of these activities remain hidden. NTL data alone cannot account for GDP values unless population density of the region is taken in the picture. Analysis by Bhandari and Roychowdhury (2011) [2] showed that unless the population density for a region is too high or too low, the NTL data is able to explain the variations in GDP and economic activity for the place. This could be due to the fact that the regions with high population density have tall skyscrapers due to lack of enough city area and thus point towards another limitation of using NTL data. Since the satellite captures images from the top, it is just able to capture the light output for the top of the building and not for all of its floors, or NTL can only account for the horizontal spread of the lights, but the vertical spread remains unaccounted for. Thus, observing a lower level of night lights as compared to the actual levels of economic activity. Similarly, for the areas with low population, NTL are not adequately captured by the current remote sensing technologies.

Doll (2008) [20] in his guide for NTL and its application mentions spatial and temporal properties of the night lights dataset which effects the efficiency of the data- *“One should be aware of the extent to which the spatial area depicted on images matches the true extent of lit area on the ground*. *Imagery from the DMSP-OLS satellite has a tendency to overestimate this parameter*, *an effect generally referred to as “blooming” (and more recently “overglow”) in the literature”*. These overestimations include large overlaps between the pixels, making the light observed in one region being recorded in more than one pixel. Errors in geolocation occur when data is transformed due to projection of the spatial positions data (collected by navigation satellite) on a 1 square km grid after correcting it for topographic variations. Atmospheric water vapor content and clouds make the light appear dimmer and more spatially diffused, reducing the quality of the captured images. Even after all these limitations, night time lights still remain one of the most favored proxy measures for GDP, especially when disaggregated GDP data is unavailable.

Particularly, in the context of using NTL to measure regional inequality, one has to be cautious about agglomeration issues. It is possible that the transition to an industrial economy prompts migration from rural to urban spaces, thereby leading to a rise in inequality across income levels. In this case, the differences in industrial specialization could be an important factor that explains differences in income across districts. On the other hand, urbanization economies could emerge in dense areas a wide and diverse range of activities, infrastructure and services. The dummy variables for capital cities and metropolitan cities might capture the agglomeration features to some extent, along with the data on population, however they might not do complete justice to the agglomeration-related issues.

Another potential source of bias might arise from the fact that the NTL data is top-coded and hence the extent of inequality rising due to the presence of the top 1% of the wealthy population, might not get entirely captured by the NTL However, since top incomes are also usually top-coded or unobserved in most cases, the extent of this bias might be negligible.

## Conclusion

We established the relationship between GDDP and NTL for the Indian sub-continent and found that NTL could indeed be used as a proxy for GDDP when controlled for the geographical and demographical factors. It follows a positive trend which is in line with our hypothesis that greater economic activity results in higher values of NTL. Though there is a presence of outliers, but it could be worked upon in the future by enhancements in NTL data measurement & recalibration techniques.

The paper further explores whether NTL data could be used in estimating regional inequality in developing countries such as India where consistent data is unavailable at sub-national-levels. The positive relationship between *Gini*_*GDDP*_ *and Gini*_*NTL*_, and, *Gini*_*Consumption*_ *and Gini*_*NTL*_, showed that NTL could be used in the absence of gross district domestic produce data for calculating income inequality. This result could be used to calculate income inequality at district-levels by using block-level NTL and population data, for which no inequality indices are available. From the heat map of Gini_NTL_, we observed that the inequality is more prevalent in the southern and western parts of India. But we also know that these are the same parts that have shown the largest domestic production growth rates in India. This could be regarded to the Kuznets hypothesis for economic development which says that as development occurs, inequality first increases till it reaches a certain threshold and then decreases. Since India still has a long way to go to become a developed nation, the Kuznets curve may be the answer for the prevalence of higher income inequality among the most fast-paced growing states. So we employed the Social Progressive Index (SPI) and Quality of Governance Index (QGI) and found an inverted-U shaped relationship with *Gini*_*NTL*_, for both SPI and QGI respectively. This further supports our hypothesis that income inequality in India might be following Kuznets curve.

The NTL data thus possesses tremendous potential for studying output, inequality, and other related measures at highly disaggregated levels and therefore should be taken seriously by policy-makers for a better understanding of the economy of a developing country like India. However, as pointed out in the paper, there are some limitations that are a feature of the nightime lights and hence usage of this kind of data and the subsequent conclusions based on this data should be done carefully.
